# Designing a stakeholder-inclusive service model for an eHealth service to support older adults in an active and social life

**DOI:** 10.1186/s12913-021-06597-9

**Published:** 2021-07-05

**Authors:** Marijke Broekhuis, Marit Dekker-van Weering, Cheyenne Schuit, Stefan Schürz, Lex van Velsen

**Affiliations:** 1 Roessingh Research and Development, eHealth group, Roessinghsbleekweg 33b, 7522AH Enschede, The Netherlands; 2 Biomedical Signals and Systems, Faculty of Electrical Engineering, Mathematics and Computer Science (EEMCS), University of Twente, Enschede, The Netherlands; 3 TSN Thuiszorg Groningen, Queridolaan 5, 9721SZ Groningen, The Netherlands; 4 National Foundation for the Elderly, Smallepad 30e, 3811 MG Amersfoort, The Netherlands; 5 LIFEtool gemeinnützige GmbH, Linz, Austria

**Keywords:** eHealth, User-centred design, Service model, Implementation

## Abstract

**Background:**

Service model design is slowly being recognized among eHealth developers as a valuable method for creating durable implementation strategies. Nonetheless, practical guidelines and case-studies that inform the community on how to design a service model for an eHealth innovation are lacking. This study describes the development of a service model for an eHealth service, titled ‘SALSA’, which intends to support older adults with a physically active and socially inclusive lifestyle.

**Methods:**

The service model for the SALSA service was developed in eight consecutive rounds, using a mixed-methods approach. First, a stakeholder salience analysis was conducted to identify the most relevant stakeholders. In rounds 2–4, in-depth insights about implementation barriers, facilitators and workflow processes of these stakeholders were gathered. Rounds 5 and 6 were set up to optimize the service model and receive feedback from stakeholders. In rounds 7 and 8, we focused on future implementation and integrating the service model with the technical components of the eHealth service.

**Results:**

While the initial goal was to create one digital platform for the eHealth service, the results of the service modelling showed how the needs of two important stakeholders, physiotherapists and sports trainers, were too different for integrating them in one platform. Therefore, the decision was made to create two platforms, one for preventive (senior sports activities) and one for curative (physical rehabilitation) purposes.

**Conclusions:**

A service model shows the interplay between service model design, technical development and business modelling. The process of service modelling helps to align the interests of the different stakeholders to create support for future implementation of an eHealth service. This study provides clear documentation on how to conduct service model design processes which can enable future learning and kickstart new research. Our results show the potential that service model design has for service development and innovation in health care.

**Supplementary Information:**

The online version contains supplementary material available at 10.1186/s12913-021-06597-9.

## Background

For an eHealth service to be implemented, one needs to engage stakeholders right at the beginning of the design process to select system functionalities that suit the user needs and (medical) context [1, 2]. Furthermore, it is important to create commitment among stakeholders, so that they willingly collaborate and have a shared vision on how the eHealth service is to be put into practice [3, 4]. There are ample methods for identifying relevant stakeholders [5–7], and models and approaches for identifying end-user and stakeholder needs and wishes for the design of an eHealth service [8–11]. However, these methods are meant for technological development processes, meaning the focus is on making sure the functionalities and look and feel of the eHealth technology are in line with the needs of the end-user(s) and to ensure uptake of the eHealth service. What is missing are insights on how the user will experience the eHealth service in daily life and the roles of the stakeholders within this experience. Furthermore, most research methods focus on the end-user(s), while giving less attention to other stakeholders that are required to implement the service in real-life. To develop a shared vision among stakeholders on the use and experience of a service, service modelling is crucial.

Service modelling, sometimes referred to as service blueprinting, describes the process of how an eHealth service can be used in an eco-system. Stakeholders are often included in the model, to highlight their roles in the service provision. For the sake of this study, we refer to stakeholders as both the main end-users, who use the technology directly (direct stakeholders), and the other parties, who are indirectly affected by the technology (indirect stakeholders) [12]. For example, when developing an eHealth technology for people with cardiovascular diseases (CVD), direct stakeholders are probably the people with CVD, and indirect stakeholders include health care professionals that are involved in the rehabilitation process of people with CVD, general practitioners, and a cardiac rehabilitation centre [13]. Service models are often graphically presented [14, 15], which makes them easier to reflect upon and to evaluate by the different stakeholders. There is no single way in which service models should be developed, but there is consensus on what they should include. A service model should show all single steps taken by the end-user in the service delivery process, as well as a holistic overview of the entire process, including background activities [15, 16]. It explains how direct and indirect stakeholders are introduced to the product or service, the manner in which they interact with the different components, and the consequences that stem from these interactions [16–18]. A detailed service model can also be useful to serve as a blueprint for developing value propositions, for optimizing the technological architecture, and for developing implementation plans. Furthermore, a service model is highly useful as a starting point for business modelling. As a service model shows the interplay between different stakeholders, it makes it easier to determine and monetize the main values for each stakeholder [19, 20].

Several studies [15, 21, 22] give examples of what service models can look like and what they should include. However, they do not describe how service models are developed, or how the perspective of stakeholders are gathered and processed. In the context of eHealth, service models are scarce [23, 24]. We lack an overall understanding of how service modelling can be applied during the eHealth service development process.

This article describes the development process of a stakeholder-inclusive service model for an eHealth service. Developing a service model is an iterative and gradual process, where one begins with a rough sketch of the basic idea. During subsequent studies, one can alter, elaborate on and improve the service model. In this article, we explain the steps that can be taken in this process and how the results of the service model design process feed back into the eHealth service development process (and vice versa). Furthermore, we show the different interim versions of the service model to illustrate how such a model matures with each round. Last, we explain the (dis)advantages of this approach in relation to other well-known models and methods on stakeholder inclusion during the development of eHealth services.¶.

## Methods

### The SALSA service

Within the Active Assisted Living (AAL) project titled ‘Supporting an Active Lifestyle for Seniors through an innovative App-based system for Fitness and Physiotherapy (SALSA)’ the aim is to develop a digital eHealth service, called ‘SALSA’, to bring older adults (55 years or older) from local communities together in active group events, both in a preventive context (senior sports groups) as in curative context (group rehabilitation therapy).

### Preparation

The starting point of the service model design process was a design brief for developing an eHealth service for support older adults in a physically active and socially inclusive lifestyle. With the latter, we mean an age-friendly environment in which older adults ‘*can cultivate social relationships, have access to resources and feel part of the community’* ( [25], p2). Three personas were created by the project team, based on existing literature and similar projects that represent the main direct stakeholders: older adults and physiotherapists. There were two personas for older adults (Marcus and Jenny) and one for the physiotherapist (Wendy). These personas are helpful to show to and discuss with the stakeholders how the eHealth service is to support them in their daily lives or work routine [26]. The personas are described in the Additional file 1: Appendix A.

### Development of the service model

**Fig. 1:**
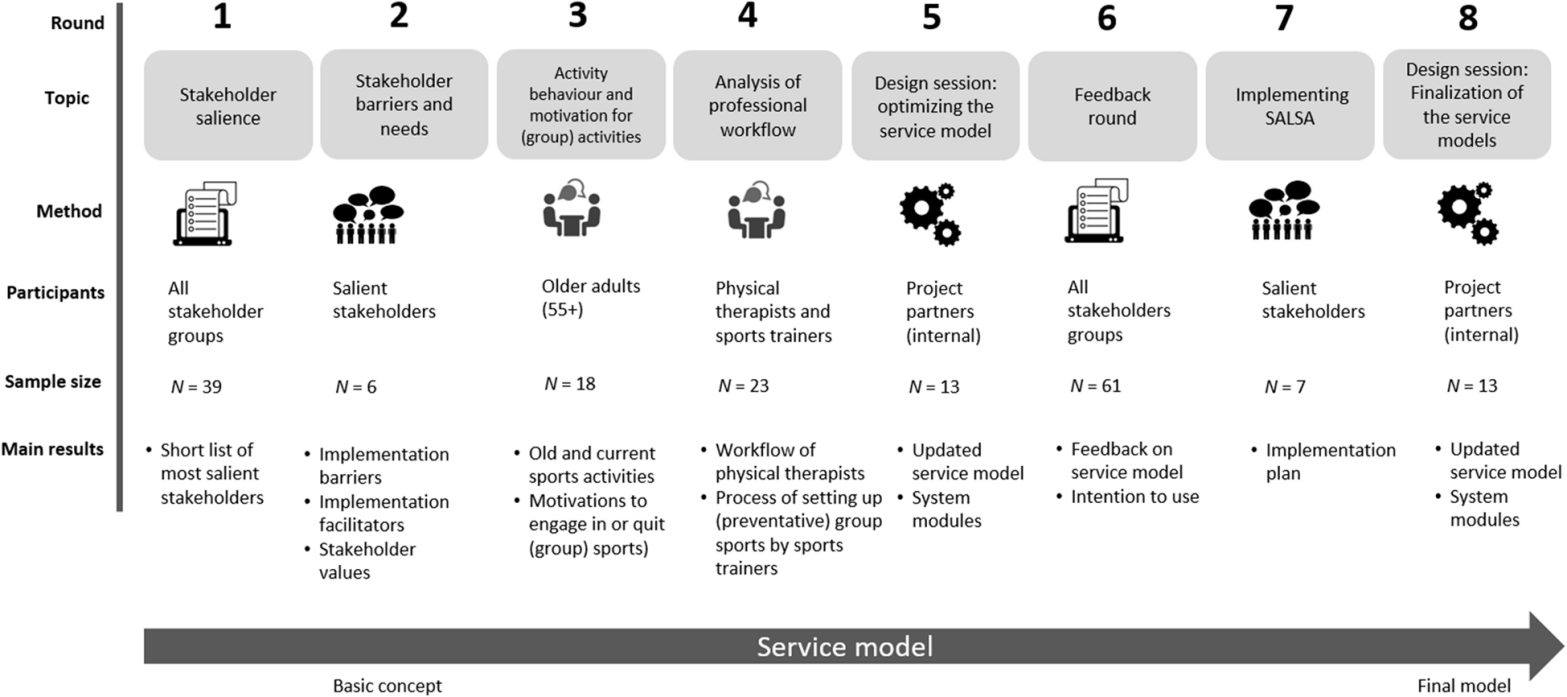
Service model design process for the SALSA service

A stakeholder-inclusive service model was developed in eight consecutive rounds (see Fig. 1), in which the service model gradually took more shape with each round. The service model was developed between May 2019 and January 2020. The interaction with stakeholders for the development of the service model took place in the Netherlands, Austria and Switzerland, as these are the countries in which the SALSA service is to be implemented. Next, we explain the methods per round.

#### Round 1

The goal of this round was to create a shortlist of the most salient stakeholders for the SALSA service, so that they could be invited for co-design sessions. For this purpose, an online stakeholder salience survey was administered among potential stakeholders in the Netherlands, Austria and Switzerland using the purposive sampling [27]: people were invited to participate if they fit one of the potential stakeholder categories that were identified beforehand. The survey was based on the stakeholder salience attributes (power, legitimacy and urgency) from Mitchell et al. [5]. After reading a short description of the SALSA service, participants were shown a list of the potential stakeholders and were asked to fill out how powerful, legitimate and urgent they believed each stakeholder was for the SALSA service on a five-point Likert scale. Power was defined as: the degree to which the following individuals or organisations have power to influence the success of putting SALSA into practice. Legitimacy was defined as: the degree to which it is correct and justifiable that we involve the following individuals or organisations when putting SALSA into practice. Urgency was defined as: the degree to which stakeholder claims call for immediate attention, when putting SALSA into practice.

#### Round 2

The next step was to identify facilitators and barriers for implementation, from the point of view of salient stakeholders. Second, we wanted to know more about their underlying values while supporting older adults with a healthy lifestyle.

A two-hour focus group was held in Enschede, the Netherlands. Participants were recruited via purposive sampling [27] in our network if they fit one of the most important stakeholder categories as identified in round 1. Participants were asked to bring a picture to the workshop of an initiative to stimulate discussion on physical activity or social inclusion among older adults (55+ years) that they deemed successful and present this at the start of the workshop. This was used as a discussion starter to elicit their values [23]. Then, the discussion moved towards barriers stakeholders experienced regarding services supporting older adults. After that, we discussed what stakeholders believed to be most important for these types of services and initiatives. Last, we presented the value proposition of the SALSA service and showed several mock-ups of the technology to gather first impressions from the participants.

#### Round 3

The main goals in round 3 were to gain in-depth insights of older adults aged 55 years or older in (1) their current (group) activities and (2) their motivations for (not) engaging in physical (group) activities. Third, we wanted to discuss the potential role of the SALSA service.

One-on-one interviews with persons of 55 years or older were conducted in the Netherlands. Recruitment took place via convenience sampling [27] via the network of one of the authors. The participants took part in a one-hour semi-structured interview in which we asked the participants about their physical activities, their health (problems), and engagement in group activities in their local community.

#### Round 4

The goal of this round was to (1) map the workflow of physiotherapists for therapy programs and to identify where in the care path the SALSA service could be of added value and (2) to gain insights in challenges that sports trainers face in setting up group sports activities for older adults.

Physiotherapists, with experience in treating older adults, took part in a one-hour interview session. Participants were recruited via purposive sampling [27] in our own network. A semi-structured interview was conducted to identify the professional workflow of treating older adults and the role SALSA could play in this workflow. A use case was selected of a typical health problem that comes with older age, namely hip injuries. Afterwards, the interviewer combined the input from both physiotherapists to create one general workflow for treating older adults with fractured hip. Furthermore, a group session was conducted with sports trainers that organize and set-up walking sports (adapted sports for older adults), during a full-day training workshop.

#### Round 5

An internal workshop was organized with the project team to optimize the initial service model and to align the service model development with the technological development of the SALSA service.

A two-hour workshop on service modelling was conducted with the project team. The participants were split into groups and each group was given a different persona (Marcus, Jenny or Wendy). They were asked to evaluate the service model from the perspective of their persona and provide suggestions on how the service model could be further enhanced. The input from the workshops was used to refine the service model.

#### Round 6

The aim of this study was to gather feedback on the service models from stakeholders, to gauge their attitudes towards the implementation of SALSA and their opinions about the different functionalities that the SALSA service offers.

A large-scale online study was conducted in the Netherlands, Austria and Switzerland. For the purpose of this study, we transformed the service model into a storyboard. See Additional file 1: Appendix B for some screenshots of this storyboard. It tells the story of Jenny, a woman of 66 years who joins SALSA Fun to meet peers for group sports activities. When she breaks her hip, she has to go to the physiotherapist who recommends her to use SALSA Health, to perform home exercises as a supplement to her regular treatment program. This storyboard was shown as a video in the online survey. The survey contained a combination of closed and open-ended questions in which the participants had to indicate if they would like to use the SALSA service and if they thought it was of value in comparison to other eHealth services that target older adults to engage in more physical or social activities. The open-ended questions were analysed to identify positive and negative factors the stakeholders mentioned regarding the SALSA service. In this article, we highlight the main results. The closed questions were analysed using descriptive statistics (means, frequencies, percentages).

#### Round 7

This study was set up to identify potential bottlenecks for implementation and to create solutions for these bottlenecks.

A focus group was set up in Enschede, the Netherlands, to discuss future implementation of the SALSA service. Participants took part after purposive sampling [27] if they fit one of the main stakeholder categories as identified in round 1. We recruited them via our network. At the start, the service model for the SALSA service was presented and discussed. Then, we presented a timeline for implementation of the SALSA services, split into three main phases: before, during and after implementation. The participants first listed implementation problems, after which discussion followed about these problems and recommendations to improve the uptake of the system. These recommendations were categorized by two researchers (MD and MB) based on whether they should take place before, during or after using the system.

#### Round 8

For the final round, we invited all members of the project team to discuss their roles and the technical components of the system within the service models.

To finalize the service models, we gathered input from each project partner on the technical components or content modules that they would bring to the SALSA service. This was done as preparation for a workshop with all project partners. During this workshop, a concept service model was presented that includes the contribution (in terms of technology or offline services) to SALSA. The workshop, which had a semi-structured character, had two goals: (1) to verify these concept service models and (2) to obtain agreement among the project partners upon roles and responsibilities.

### Data analysis

A mixed-methods, applied research approach was used, consisting of a combination of online questionnaires, in-depth interviews, focus groups and workshops. In applied research, the goal is to use knowledge and practice towards a specific purpose [28] and to focus on actionable outcomes which affect how data analysis is conducted [29]. For each round, the goal was to gain answers to the questions or issues addressed, which was subsequently used as input for the next round. For quantitative data, descriptive statistics were calculated. For the qualitative data, audio recordings were made of each session and were transcribed and analysed using deductive framework analysis [30, 31] based on predefined topic areas. For each round containing qualitative data collection, audio recordings were made and transcribed. One researcher (MB) familiarized herself with the data. Then, the data was coded and grouped based on overarching categories. We used the predefined topic list to create an initial list of the main categories. Subcategories were created by using (groups of) codes. This category-overview was then handed over to a second researcher (MD) who also coded another subset of the data. The researchers MB and MD cross-checked each other’s categories and differences were solved to agree on the category-overview. We reported the results following the COREQ standard [32].

## Results

### Round 1

A total of 39 participants completed the stakeholder salience survey. The scores for each stakeholder group can be viewed in Table 1. As the data was not normally distributed, we opted for non-parametric analysis methods. The following stakeholders had the highest median scores and thus, were identified as the most salient stakeholders for the SALSA service: older adult (55 years or older), general practitioner (GP), regional health initiatives, physiotherapist, rehabilitation centre, geriatric medical specialist, sports coach. There is an almost equal distribution in clinical and non-clinical stakeholders. For the next rounds, we invited representatives from these stakeholder groups.

**Table 1:** Mnd, IQR of stakeholder salience attributes (power, legitimacy, urgency) per stakeholder group

Stakeholder	Participant?	***N***	Power		Legitimacy		Urgency	
			***Mdn***	***IQR%***	***Mdn***	***IQR%***	***Mdn***	***IQR%***
Older adult (55+)	Yes	13	3	2, 5	5	3.5, 5	4	3, 5
Informal caregiver	Yes	10	3	2, 4	3.5	2, 4	3	2, 4
General practitioner	Yes	3	4	3, 4	4	3, 4	4	2.25, 4
Nurse practitioner	No	n/a	4	3, 4	4	3, 4	4	2.75, 4
Community nurse	No	n/a	4	3, 4	4	3, 4	4	2.75, 4
Municipal health initiatives	Yes	2	3	3, 4	4	3, 4	4	3, 4
Physiotherapist /occupational therapist	Yes	9	4	4, 4	4	4, 4.25	4	4, 4
Management of physical therapy practice	Yes	2	3	2, 4	4	3, 4	3	2, 4
Health insurance company	No	n/a	3	2, 5	3	2, 4	3	2.75, 4
Rehabilitation centre	Yes	2	4	3, 5	4	4, 4	4	3.25, 4
Geriatric medical specialist	No	n/a	4	3, 4	4	3, 4	4	3, 4
Home care organisation	Yes	1	3	2, 4	3	2, 4	3	2, 4
(National) senior organisation	Yes	4	3	2, 4	3	2, 4	3	2, 4
Sports club	No	n/a	3	3, 4	4	3, 4	3	2, 4.25
Sports coach	No	n/a	3	3, 4	4	3, 4	4	3, 4
Local activity coordinator	Yes	2	3	2, 4	3	2.25, 4	3	3, 4
Manager of sports club/association	Yes	1	3	2, 3.25	3	2, 4	3	2, 3.5
Senior residences/retirement homes	No	n/a	3	3, 4	3	2, 4	3	2, 4
Social services by volunteers	Yes	1		1, 3	2	1, 3	2	1, 3
Decision maker municipality	No	n/a	3	2, 4	3	2, 4	3	2, 4

### Round 2

Six people attended the focus group: two older adults, a sports trainer, one physiotherapist, one innovation manager from a care institute and one innovation manager from a rehabilitation centre. GP’s and geriatric medical specialists that were invited did not respond or did not want to take part in this workshop. The main topics discussed in the focus group were: facilitators and barriers for successful initiatives for older adults, stakeholder values, and the potential role of technology. We will next explain each topic.

#### Facilitators

The participants mentioned six facilitators for successful initiatives for older adults. These services or initiatives should enhance (1) social inclusion (feeling part of a group and social interaction), (2) participation in society (interaction with other groups, like collaborations between care homes and schools), (3) mobility (being able to visit other places), (4) enjoyment (the initiatives should be fun and enjoyable), (5) patient empowerment (initiatives should stimulate older adults to gain more control over their health) and (6) better care for older adults (initiatives should lead to better care).

#### Barriers

Five implementation barriers regarding the SALSA service were identified: costs, access to users with low Socio-Economic Status (SES), segmentation of the healthcare sector, health information, and overprotection of health care providers and informal caregivers. Table 2 shows these barriers and provides recommendations from participants to overcome these barriers.

**Table 2:** Implementation barriers and recommendations

Implementation barrier	Recommendation
Costs	The costs for the SALSA service should be kept at a minimum for older adults with lower SES who cannot afford full healthcare or sports classes.
Access to low SES users	Stakeholders believed that people with low SES that would benefit a lot from a service like SALSA. But reaching these people is challenging. To improve access to this group, one should make use of social workers, friends, family, or a physical therapy practice.
Segmentation of the healthcare sector	The healthcare sector in the Netherlands is strongly segmented, with each segment having its own financing structure. This makes it difficult to find common ground among organisations within and outside the healthcare sector, as their interests lie (too) far apart for an integrated approach.
Health information	Many older adults mistake ‘exercising’ for sports-related activities only, while health professionals also mean daily chores, like gardening, cooking, cleaning. Thus, even if older adults attend a weekly sports class, their daily physical activity levels are often low. More information about what it means to be active and about activity guidelines is necessary for this group.
Overprotection of health care providers and informal caregivers	Health professionals are trained to care for their clients when they are hospitalized. This often results in patients being inactive for most part of the day. However, the patients’ recovery process would benefit by staying physically active and do simple tasks like getting coffee. Informal caregivers are subject to the same paradox. More information for health professionals and informal caregivers is necessary about supporting patients with their recovery by helping them to perform daily tasks independently.

#### Values

From the discussions, three values emerged that participants believed were most important for supporting older adults with a healthy lifestyle. First, the attention should be on how society in general can *mutually help and support* one another. Second, there is a general feeling that there should be much more *interaction and relationships within society.* Third, people should be more *altruistic*. The sports trainer explained that most sports clubs ask rent for volunteer senior sports groups to use their fields and facilities, while their members often have a few drinks in the canteen (which is, in his opinion, a more profitable income than the rent for fields and facilities). This forces him to ask his members for a fee, making the training sessions less accessible for people with lower incomes.

#### Potential role of technology

It became clear that stakeholders believed that the social network of older adults could play a large role to find those people that would benefit from a service like SALSA and to motivate and encourage them to become or remain active, both physically and socially. Because costs for users could potentially be a major barrier for older adults to use the SALSA service, we should try to find ways to implement the service in such manner that the costs for continuous development, support and maintenance of the website and app are not paid for by membership fees of older adults. For such a service to be successful, the stakeholders emphasized how the focus should not be solely on increasing physical activity levels, but more on strengthening social bonds and contacts between older adults and, potentially, between people of different age groups.

**Fig. 2:**
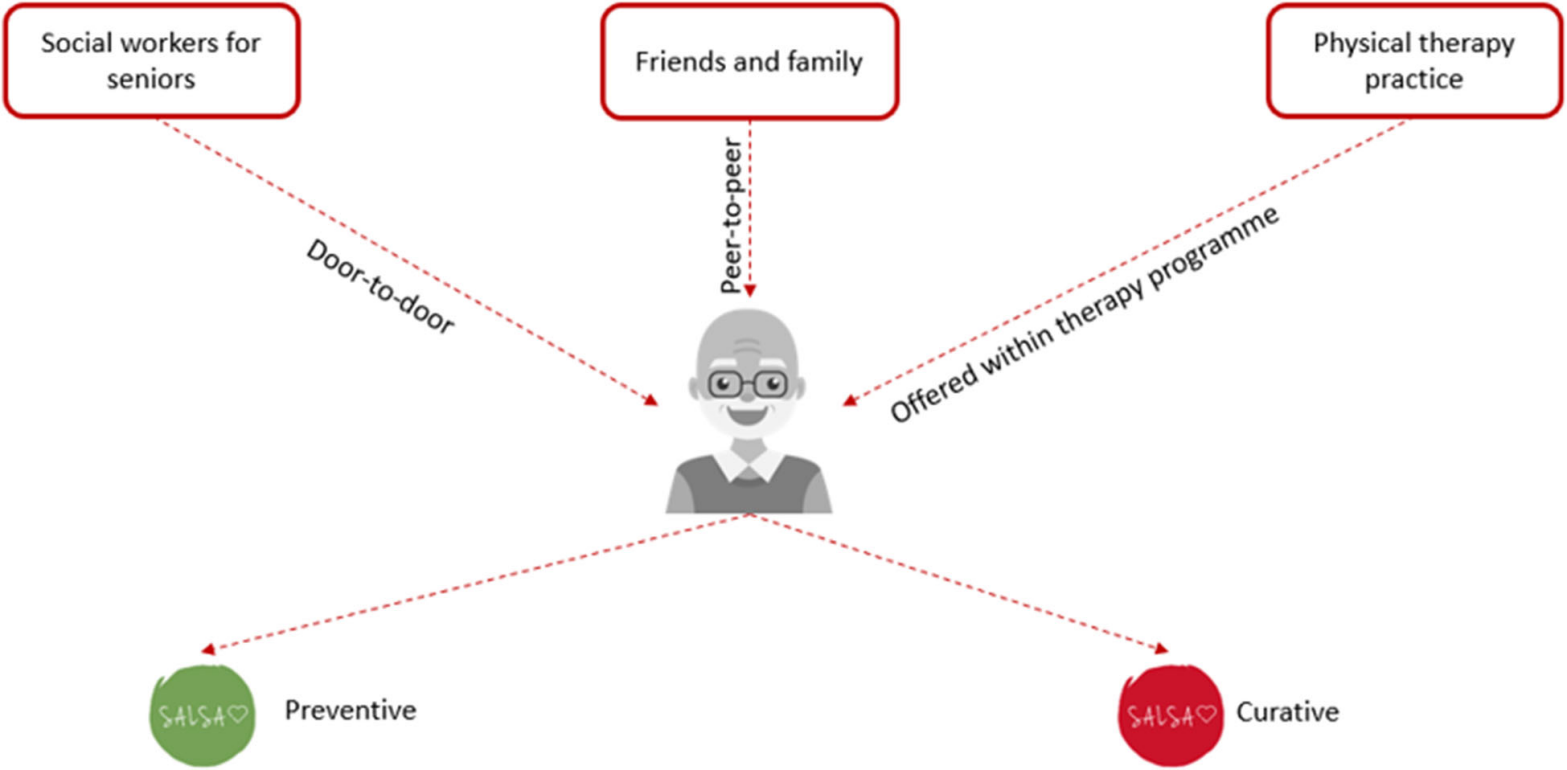
Initial sketching of the service model

Figure 2 shows a first sketch of the service model. Based on discussions with the stakeholders, we identified some points for first contact. Also, we made a distinction between preventive purposes of the SALSA service (like senior sports activities) and curative purposes (for older adults who need or are following physiotherapy).

### Round 3

Eighteen people (seven male and eleven female) of 55 years or older participated. The average age of the participants was 71 years. Major topics that emerged in these interviews were (1) old and current physical activities of older adults, (2) motivation for (group) sports of older adults, and (3) the implications for implementation of the SALSA service. We will next explain each topic.

#### Old and current physical activities

Participants’ current physical activities included a combination of individual and group sports, like combining an elderly sports class with TV fitness programs. The majority of the participants took part in approximately the same number of sports activities (between 1 to 3 different sports), although the type of sports could change over time. Participants often stopped exercising when their health deteriorated or when there was a decline in members of a sports class. Instead of joining new group, they often decided to stop exercising because they had difficulty finding new groups. Some participants indicated they did not want to socialize with new people anymore. Other participants, that were enthusiastic to take part in group sports, said it would motivate them to go to the sports class.

#### Motivation for (group) sports

Participants liked the combination of individual exercises with sports classes. Social interaction during or after a sports class is important. They indicated that they would go to sports classes more frequently if they were recommended by family members or friends and if the activity was in their local neighbourhood. There should also be an option to exercise at home, since mobility is often a problem for them. Participants indicated that they did not want to change groups frequently. They wanted to stay with the same group as long as possible. Therefore, they preferred to join sports classes that they can continue to do even when their health deteriorates (like walking or swimming).

#### Implementation of the SALSA service

The participants believed that employers, fitness clubs, physiotherapists, and community centres can play a large role in promoting the SALSA service. For older adults to use the system for a longer period of time, the costs should be low and the system should be usable for people with low digital skills. As they liked the combination between group and individual sports, this should also be reflected in the system; older adults should be able to use the system as a standalone tool for home exercises and to use it to find and join group sports. For successful implementation of the SALSA service, the participants mentioned three main aspects that should be considered: costs, digital skills and type of activities (e.g. activities that are fitting for older adults and that they can continue to do even when their health slowly deteriorates, like cycling, swimming, or walking). Physical therapy practices were mentioned as a gateway to find older adults. The Central Bureau for Statistics (CBS) in the Netherlands showed that between 2017 and 2019, approximately one-third of people who are 55 years or older visit at least once a year a physio-or occupational therapist [33, 34]. This makes physiotherapy practices an interesting option to attract older adults, especially those with lower incomes who are more difficult to reach, as mentioned by the stakeholders in round 2.

**Fig. 3:**
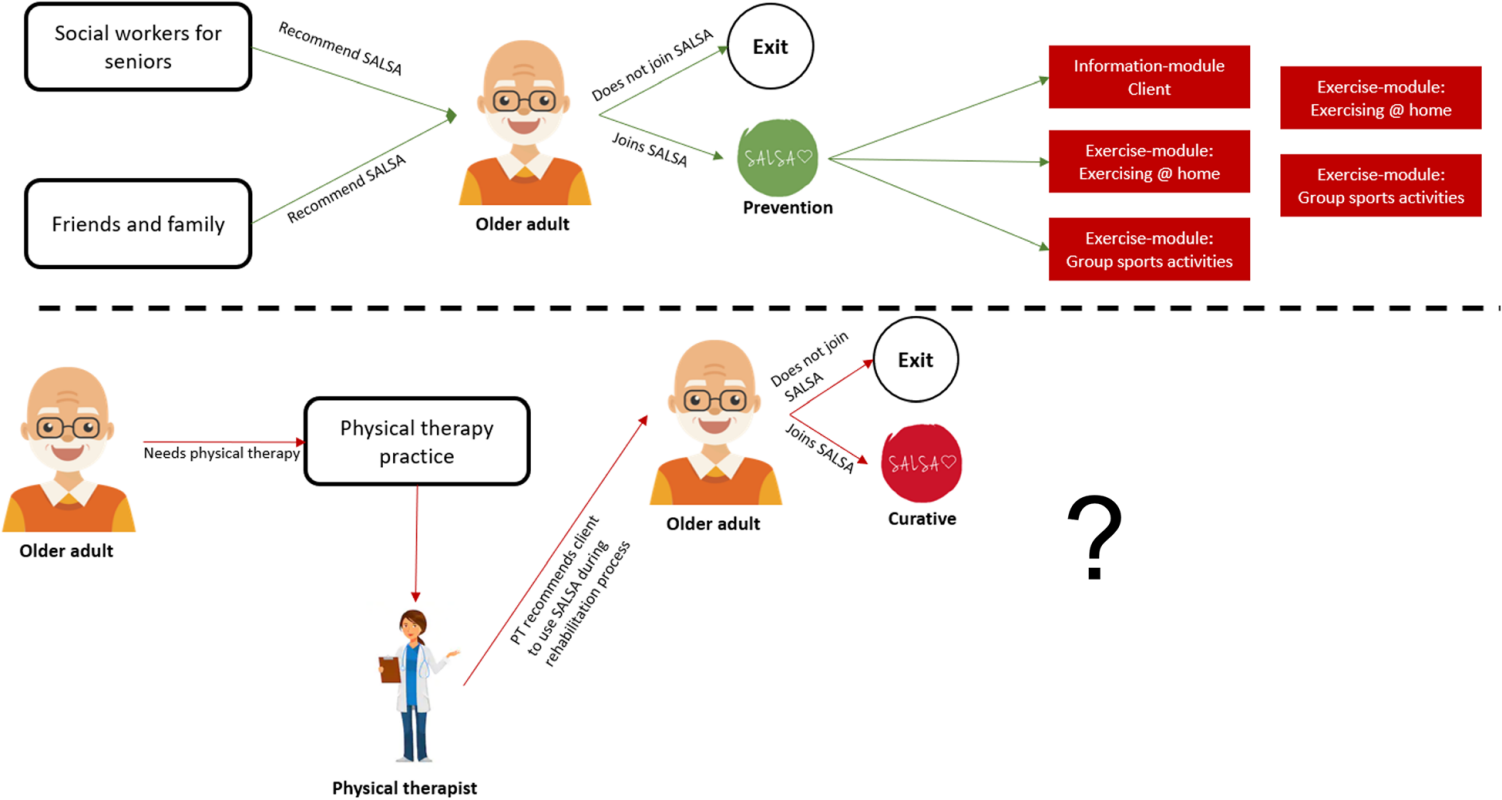
Second sketch of service model

Figure 3 shows the second sketch of the service model. We have made a clear distinction between people who want to use SALSA to meet peers at senior sports activities or to exercise at home, and people who want to use SALSA within physiotherapy treatment programs. How the SALSA service could be integrated within physiotherapy treatment is yet unknown (hence the question mark). Therefore, for the next round we will focus specifically on physiotherapists, to gain insights in the workflow and treatment of older adults.

### Round 4

Two physiotherapists and 21 sports trainers took part in this round. The results are shown per stakeholder group (physiotherapists and sports trainers).

#### Physiotherapists

The general workflow for physiotherapists can be seen in Fig. 2. Patients are normally referred to a physiotherapist by his or her general practitioner or a medical specialist. During the first consult, the physiotherapist sets goals with the patient and defines the treatment program. During treatment, the physiotherapist advises patients to do activities at home, like exercising, fitness or walking, in addition to the therapy program. Towards the end of the program, the physiotherapist can offer group training sessions for fitness and falls prevention. When treatment has ended, the physiotherapists advise patients to return when pain or symptoms return. Also, they are given an exercise book with exercises to do at home. Patients are advised to continue being active, doing sports and to walk as much as possible. In case the patient has made little to no progress in recovery, he or she is referred back to the general practitioner.

The SALSA service can support the therapy-workflow especially in the beginning of and during treatment. At the start of the therapy, the physiotherapists indicated that the SALSA service could support them in providing health information about the treatment and health condition to their patients. During treatment, monitoring is important, to see if their clients perform the exercises at home and if they perform these correctly. At the end of the treatment, the physiotherapist can use the SALSA service to give his or her client additional exercises to do at home or, if the client is physically fit enough, recommend some preventative group sports activities for seniors in the neighbourhood.

#### Sports trainers

Discussions with sports trainers resulted in four possibilities for the service model. First, the SALSA service should support recruitment of new members. For sports groups and classes to survive, it is vital that they continuously recruit new members. This can be done by introducing the SALSA service to existing sports groups and from there, make use of word-of-mouth advertising and the network of trainers and members. This way, people can be made aware of the SALSA service and of the sports groups that are offered via the service. Second, there should be an option for users to switch between preventive sports activities and health (physiotherapy) exercises. When members get an injury and need physiotherapy they can use the SALSA service to support their recovery process and vice versa (join regular sports classes after physiotherapy). Third, sports trainers indicated that they often have little experience with people with health conditions, like cardiac diseases or back pain. Therefore, the SALSA service can support training sessions and education among sports trainers on how to set up training sessions, making these sessions suitable and accessible for people with (chronic) illnesses and learn what are important signs that indicate that older adults are not well. Last, for retaining members it is important to ensure stability of the sports groups. Social inclusion was mentioned as a crucial aspect, to make people feel part of a group and support social interaction. Also, when people are not able to join the sports class anymore, there should be options for them to attend social events and gatherings, related to sports clubs.

From this round it became clear that physiotherapists do see the potential value of the SALSA service to be used within their therapy programs. Sports trainers have quite different needs. They need support and information about setting up engaging training sessions for their members and prefer some education on (age-related) health conditions. For them, finding and recruiting new members is always an issue, so it would also be beneficial if the SALSA service can help them with this problem. For the next rounds, it was important to investigate how to integrate the needs of both groups into one service.

### Round 5

Thirteen people of the project team took part in this workshop. During this workshop, in which also the results from the previous rounds were discussed, it became clear that there are two different use cases for the SALSA service. For preventive purposes, there is a main role for sports trainers that are both direct and indirect stakeholders. They are vital for setting up senior-appropriate sports training sessions and recruiting older adults for sports classes. In contrast, for rehabilitation purposes, the SALSA service should include exercises and group training sessions that are recommended by physiotherapists and that can be adapted towards the patient’s recovery process. Therefore, there was a consensus that there should be two separate systems: one for prevention, to promote healthy ageing (SALSA Fun), and one specifically designed as a support tool within rehabilitation therapy (SALSA Health). These two systems are not mutually exclusive. Users can go back and forth between them.

SALSA Fun is a platform for older adults to find group sports related activities, specifically for older adults within their local community. SALSA Fun supports older adults in receiving tailored information about their health and about the positive aspects of exercising, or staying active in daily life. Furthermore, SALSA Fun includes a knowledge module for sports trainers to learn more about setting up training sessions and sports events for older adults and to learn from each other.

SALSA Health is meant for physical therapy or rehabilitation purposes. It is an app-based system for exercise videos and exergames and includes a patient management module for physiotherapists. The therapists can use SALSA Health to send their patients exercises they can do at home (exercise videos or exergames).

**Fig. 4:**
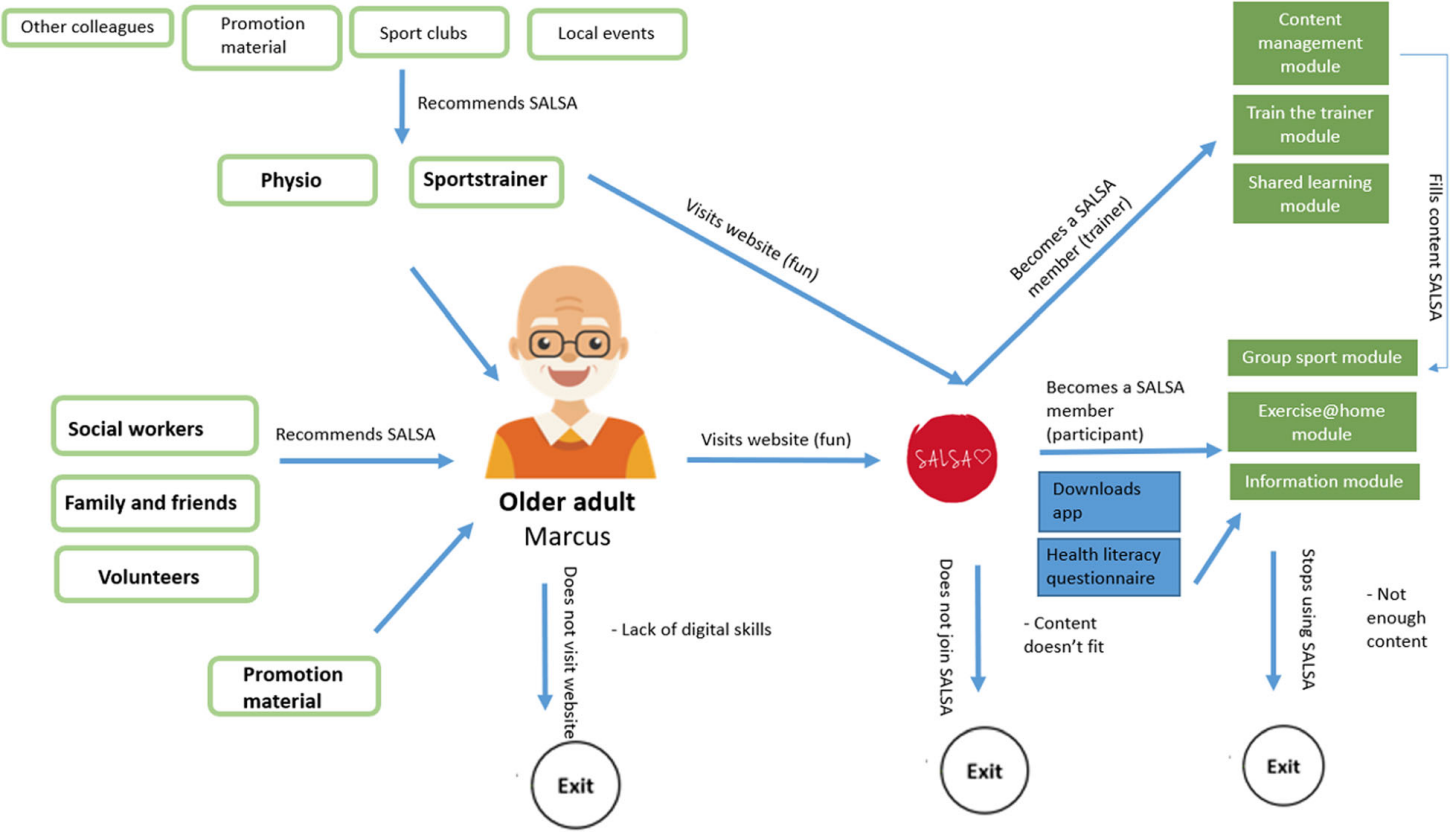
Sketch of SALSA Fun service model

**Fig. 5:**
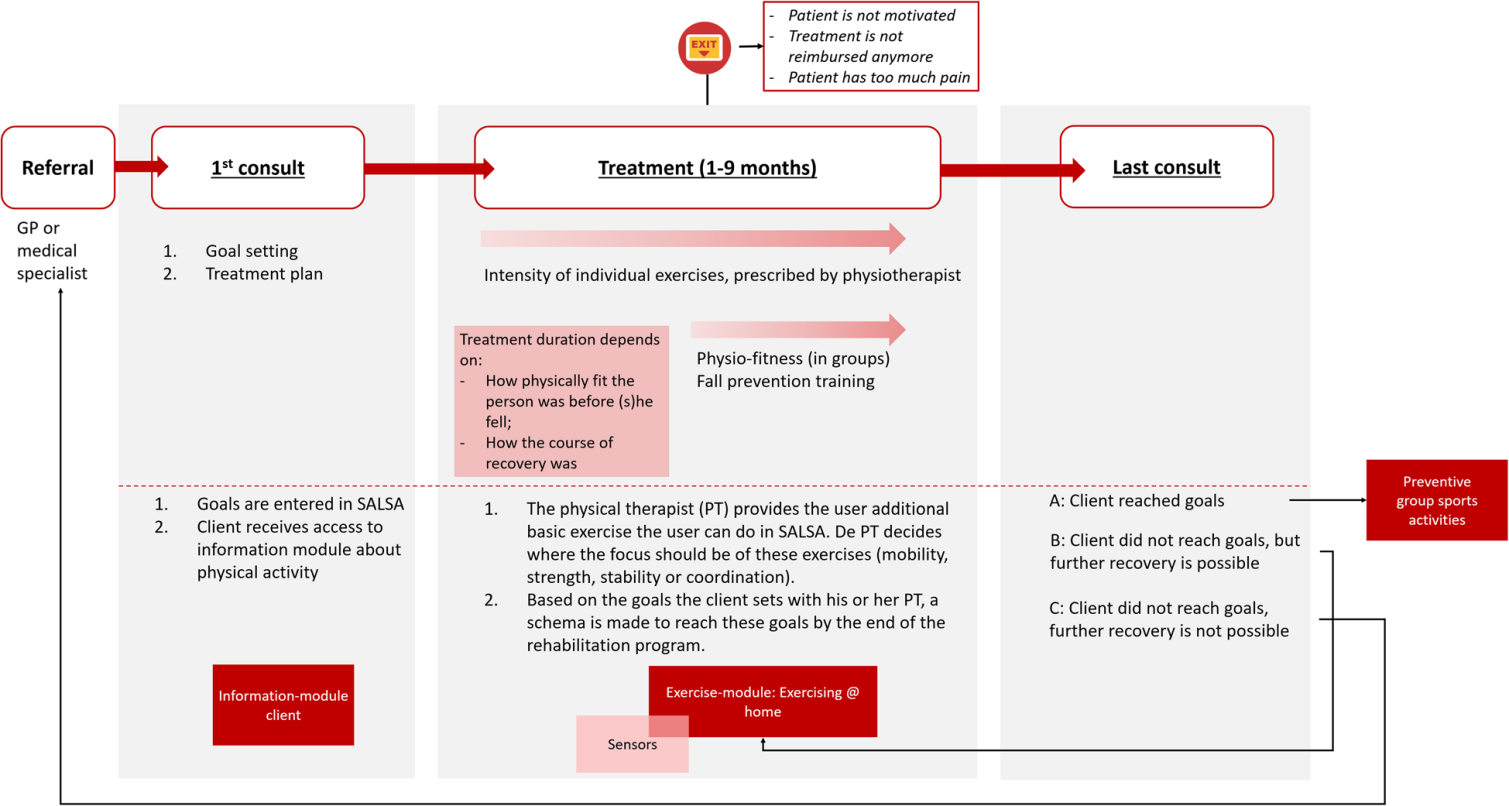
Professional workflow of physiotherapists

As it was decided that there would be two separate systems within the SALSA service, it also made sense to develop two service models, since the systems will be implemented within different contexts and include different stakeholders. The service model for SALSA Fun is centred around sports clubs and trainers to engage older adults in an active and social lifestyle within the local community (Fig. 4). The second service model should show how SALSA Health is to be implemented within physical therapy and rehabilitation programs, based on the workflow of physiotherapists (see Fig. 5).

### Round 6

A total of 61 participants filled out the complete survey. Table 3 shows the participant characteristics. Control questions revealed that 58 out of the 61 participants (95.1%) could see the video clearly and had no problems understanding the content. For showing the main results, we split the results between the stakeholders that are end-users (older adults and informal caregivers), stakeholders related to SALSA Fun (senior network association, municipal health service, sports trainer, sports association) and stakeholders related to SALSA Health (physiotherapist, nurse, manager physical therapy practice, rehabilitation clinic, manager home care organisation).

**Table 3:** Demographics per stakeholder group

Stakeholder category	***N***	***Av. age***	***Male%***	***Female %***
Someone of 55 years or older	35	68	57.1%	42.9%
Informal caregiver	5	69	20%	80%
Physiotherapist	9	45	33.3%	66.7%
Senior network association	2	75	100%	0%
Nurse	2	61	0.0%	100%
Municipal health service	2	61	100%	0%
Manager physical therapy practice	1	44	100%	0%
Sports trainer/coach for seniors	1	71	100%	0%
Rehabilitation clinic	1	51	0%	100%
Manager (home) care organization	1	48	100%	0%
Manager sports association	1	65	100%	0%
GP	1	48	100%	0%
Total	61	59	54.1%	45.9%

#### Stakeholders as end-users

This group consisted of 40 participants. Older adults and informal caregivers especially liked to be able to do exercises at home, to meet new people, and to receive health information from their therapist (see Table 4). 20 (50%) stakeholders believed they would use the services, 11 (27.5%) participants were in doubt and nine (22.5%) participants said they would not use it. The stakeholders especially saw the potential to stimulate peer contact and physical activity. They believed the service is quite usable for home use and liked it that the user seems to be in control. The most frequently mentioned negative comment was that the system seemed less suitable for older people, because of their lower digital skills. Also, they believed that the service should not be too dependent on the involvement of health professionals, in order to be implemented successfully.

**Table 4:** Preferences for service components by stakeholders who are end-users

Service components (for older adults)	SALSA Fun or Health	Preference (***N,*** %)
Exercise programs I can do by myself at home	Fun / Health	26 (65%)
Meeting new people of similar age	Fun	18 (45%)
Receiving health information and exercises from my physical therapist	Health	18 (45%)
Learning about 55+ activities in my neighbourhood	Fun	14 (35%)
Registering for 55+ activities in my neighbourhood	Fun	11 (27.5%)
Playing the exercise games, Exergames, as part of physical therapy	Health	6 (15%)
Starting my own groups	Fun	4 (10%)

#### Stakeholders related to SALSA Fun

This group consisted of six participants. They especially liked the option for SALSA Fun to learn more about setting up sports classes for senior participants (see Table 5). Also, the functionality to provide exercises for performing at home and to promote the sports classes they offer were very well liked. The functionality for health information distribution was chosen by only one participant. Five out of the six participants (83.3%) would like the SALSA service to be implemented in their region or organization and 12 (57.1%) stakeholders believed it would be of added value in comparison to other eHealth services that support healthy ageing. These participants liked how the SALSA service is a one-stop-shop solution, how it can improve communication and awareness among older adults to exercise, and to connect different (sports) providers. However, they expressed the fear that older adults would not be able to use the system because of their low digital literacy, that it remained difficult to reach older adults, and that data privacy is important.

**Table 5:** Preference for service components by stakeholders

Functionalities	SALSA Fun or Health	Preference (***N,*** %) of SALSA Fun stakeholders	Preference (***N,*** %) of SALSA Health stakeholders
For health professionals: to provide my clients additional exercises they can do at home	Health	3 (50%)	12 (80%)
To communicate and exchange information among health professionals and (sports) trainers	Fun / Health	2 (33.3%)	8 (53.3%)
For health professionals: to provide my clients additional health information	Health	1 (16.7%)	9 (60%)
To promote 55+ sports classes that I or my company organize	Fun / Health	3 (50%)	6 (40%)
For (sports) trainers: to learn more about setting up and organizing senior sports classes and training sessions	Fun	4 (66.7%)	4 (26.7%)
For (sports) trainers: to communicate with group members	Fun	2 (33.3%)	4 (26.7%)
For health professionals: to monitor if their clients perform exercises at home	Health	1 (16.7%)	2 (13.3%)

#### Stakeholders related to SALSA Health

This group consisted of 15 participants. The majority of these stakeholders liked the option to provide their clients additional exercises they could perform at home (see Table 5). In addition, the functionalities to provide health information to their clients and to exchange information between health professionals and sports trainers were frequently chosen. Interestingly, this last option was not as much preferred by the stakeholders related to SALSA Fun. The functionality to monitor the exercise behaviour of their clients at home was selected by two participants. Nine participants (60%) would like the SALSA service to be implemented in their region or organization and 7 (46.7%) stakeholders believed it would be an added value in comparison to other eHealth services that support healthy ageing. These stakeholders liked how the SALSA service could be a means to link organisations and people with each other and by this, improve communication and information exchange between these different parties. Negative comments were, similar to the older adults, focused on the fear that older adults would not be able to use the system because of their low digital literacy. Furthermore, they mentioned that these types of systems are difficult to integrate in the healthcare sector because (1) health professionals receive no financial compensation for the hours they put in the SALSA system, (2) the professional confidentiality that makes it difficult exchange health information with other parties, and (3) there is no connection between the SALSA service and electronic health records.

Overall, the SALSA service, both Fun and Health, are well received across all stakeholders. While the participants liked what they could do with the system, they did identify new implementation obstacles that need to be addressed. Therefore, in the penultimate round, emphasis should be placed on creating an implementation plan for the uptake of the SALSA service in daily life.

### Round 7

Seven people took part in this focus group: two older adults, one sports trainer and four physiotherapist. We highlight the main results per phase (before, during and after implementation). Before implementation of the SALSA service, municipalities, sports organisations and physiotherapists need to be acquainted with the SALSA service to (1) promote the upcoming service among potential end-users and organisations that could partake in the service, (2) inform physiotherapists about the SALSA service and offer training sessions or trial periods (SALSA Health), (3) collaborate with sports trainers to create sufficient content (SALSA Fun), and (4) conduct usability tests to ensure user-friendliness and accessibility of the system for older adults. From a technical point of view, physiotherapists would also like to see a technical integration of the SALSA service with their Electronic Patient Records. They did not want to use yet another system, with different login accounts.

When the SALSA service is up and running, there should be a helpdesk in place, that users can contact when experiencing problems. The physiotherapists wanted monitoring options and to have their own account of the SALSA service for their practice, in order to know which patients are using the service and to have control over what is offered to their patients. The sports trainer believed the SALSA service needed to be adopted by existing sports clubs or senior associations. Then, their members could invite other people and recommend the system to their friends and family. Thus, promoting it via word-of-mouth advertising within a local community. A moderator for each organisation was considered crucial for managing group activities.

After a fixed period of time, older adults and physiotherapists especially shared an interest in information about the results of using the SALSA service. Older adults were interested in the number of participants and sports groups or events. Physiotherapists wanted to have insight in how many clients participated and whether this system also helps to attract new clients. Clear communication about the impact and added value of the system was considered vital for upscaling the SALSA service after the end of the AAL SALSA development.

Now that we know what to consider when implementing the service, we needed to finalize the service model, based on the results of the last two rounds and the technical progress of the system.

### Round 8

This workshop with the project team led to several adaptations to the service models, mostly fuelled by requests that would increase the exploitation potential of the service.

**Fig. 6:**
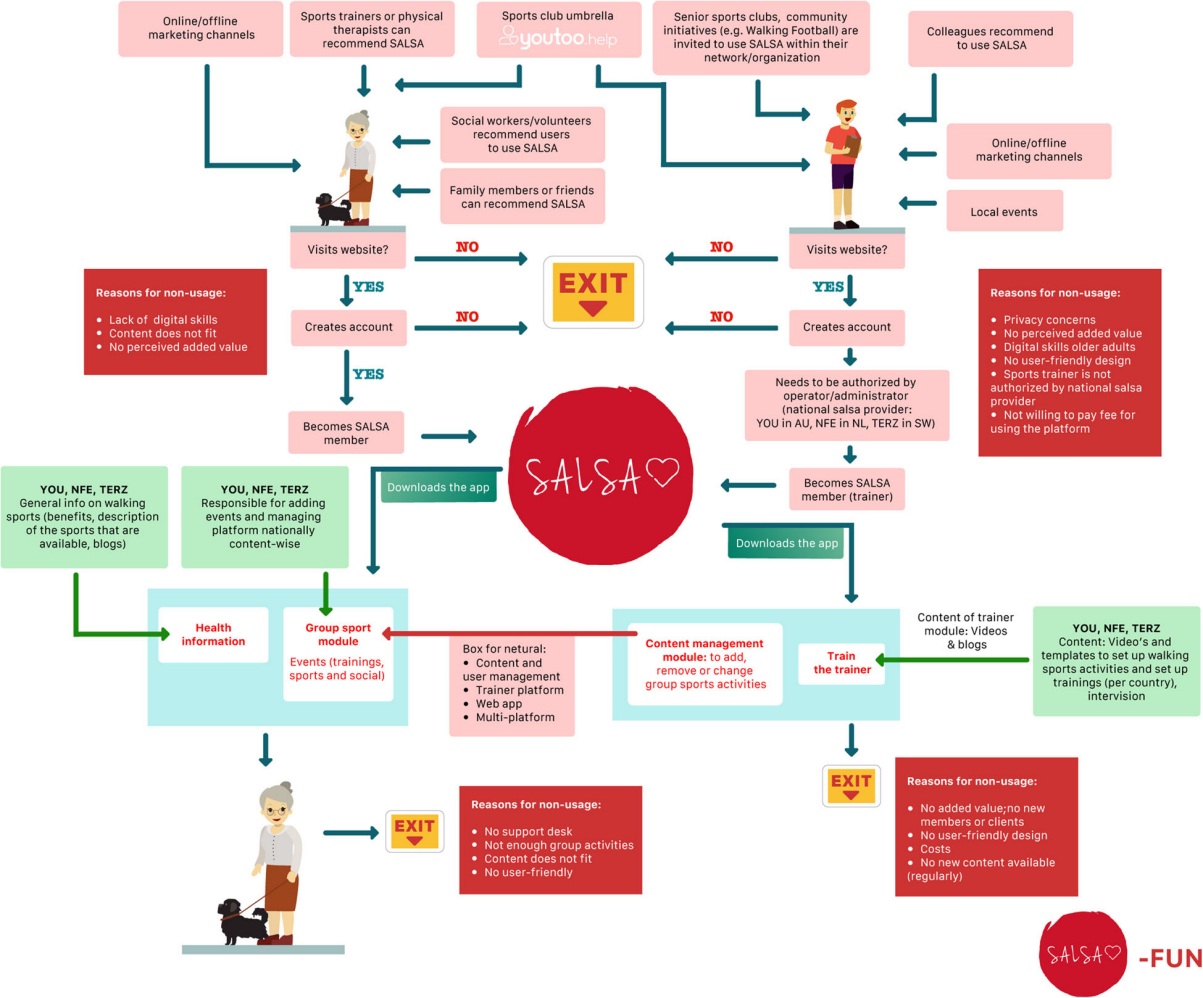
Final service model for SALSA Fun

For SALSA Fun, it was decided that sports trainers should be designated by national SALSA providers (a role taken on by project members). Sports trainers, rather than older adults, would be the purchasers of the SALSA platform. There will be no ‘exercise at home module’ for SALSA Fun, as this functionality better aligns with the value proposition of SALSA Health. Finally, it was decided that one partner per country would act as the national SALSA provider, thereby being responsible for content management and organizing meet-ups and events, such as trainer events. Figure 6 shows the final service model for SALSA Fun.

**Fig. 7:**
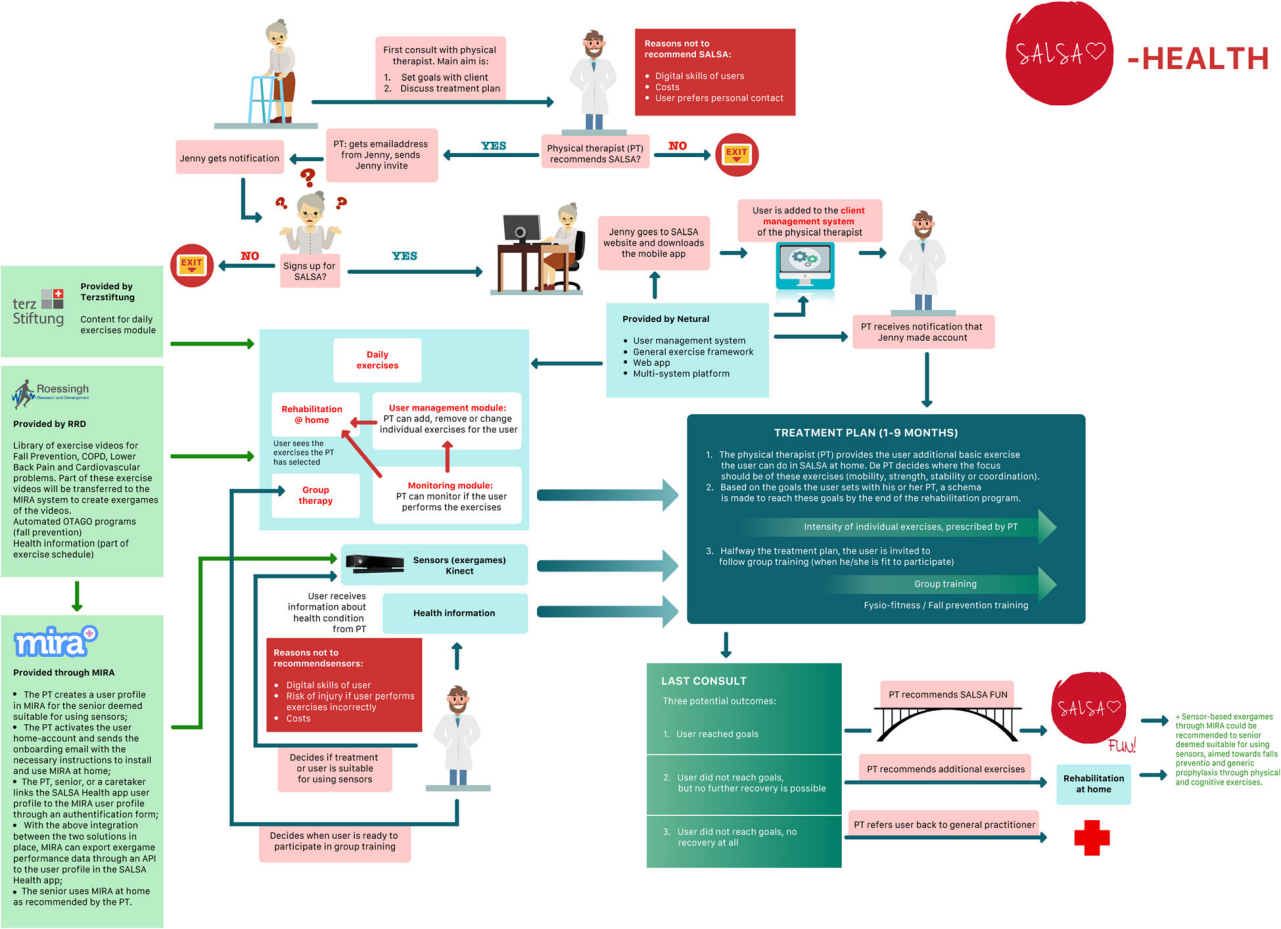
Final service model for SALSA Health

For the case of SALSA Health, several final adjustments were made to the service model. First, during the onboarding process, the therapist will, so it was decided, take the initiative in inviting clients for SALSA Health during a face-to-face consult. Then, the therapist provides the client with log-in credentials by sending an e-mail invitation via the system. Figure 7 shows the final service model for SALSA Health.

## Discussion

In this article, we described the development of a stakeholder-inclusive service model for supporting older adults in an active and social life using eHealth. Table 6 summarizes our approach and explains the activities, outcomes, people involved and suitable methods per phase that one could employ.

**Table 6:** Six-phase process of service model design

Phase	Activity	Outcome	People involved	Suitable methods
1	Identification of relevant stakeholders and determining their salience	Short-list of most important stakeholders	All relevant stakeholders for the eHealth service	Stakeholder identification [6] and salience analysis [35]
2	Acquiring in-depth information about main stakeholders	Stakeholder values, needs and their perception of the potential role of the service	The shortlist of most important stakeholders (as identified from phase 1)	Stakeholder value elicitation [36, 37] and concept validation [38]
3	Creating initial service model	Draft version of the service model	The project team (optionally: also include direct stakeholders)	Co-design/co-creation workshop [39, 40]
4	Optimizing the service model	Recommendations to improve the service model	All relevant stakeholders	Storyboards [41], paper prototypes [42]
5	Developing an implementation strategy	Implementation plan	Shortlist of stakeholders	Stakeholder workshop [43]
6	Finalization of service model	Final version of the service model	Project team (optionally: also include direct stakeholders)	Co-design/co-creation workshop [39, 40]

This approach starts with the identification of the most relevant stakeholders, after which different rounds of service model design and redesign follow. Applying an iterative approach in service modelling is paramount, so we have found. In the development of the SALSA service model, sports trainers were initially not targeted as stakeholders. However, during the process it became evident that they in fact were so, especially for SALSA Fun. From that point on, they were included for the remaining part of the service model design process. The discovery of new important stakeholders is one illustration of the added value that iterative service modelling can bring to the development process of eHealth services. Next, the approach stresses the use of creative methods (e.g., co-design workshops) to create the service model. This way, (potential) stakeholders can create a service model that is both useful and realistic, while it also improves the acceptance of the final service among these players. Recently, we have seen publications of more and more creative or playful methods for eliciting stakeholder input [44, 45]. The resulting service model can fulfil multiple goals. It can drive functional design of technology, can be the basis of an implementation plan, and can serve the marketing strategy towards different target groups.

### Comparison with literature

There is a growing need to reassess health services to provide better care and lower costs for healthcare. Researchers are becoming increasingly aware about the potential that technology has for optimizing health services, as these two are strongly linked [46]. There is a growing stream of research on integrating technology and service innovation for creating new value propositions and better customer value [47]. Service innovation is considered from the perspective of management, customer and technology [48, 49]. Service blueprinting is considered an important instrument to foster the process of service innovation. However, the full potential of service modelling (including the end-user perspective, focus on customer experience) is not yet unlocked as service models are often drafted from a managerial perspective. This makes them less detailed in terms of customer interaction, experience, technology functionalities, and environment [47]. We are of the opinion that these aspects can be quite easily integrated by adopting an all-round stakeholder perspective, as this study illustrates.

One could argue that our service model design process shares a resemblance with user-centred design (UCD) processes [50, 51]. While this is to some extent true with regard to the steps (identify needs, understand context and requirements, produce design solutions and evaluate these solutions), service model design does not focus on designing a user-friendly eHealth technology. Rather, it aims to create an understanding and shared vision of all relevant stakeholders on how the system will be used in daily life and to tackle potential implementation barriers early in the development process. As such, where UCD is primarily concerned with the end-user, service modelling processes (such as ours) take a broader view and focus on the end-user and the other stakeholders.

Another comparable model is the Centre for eHealth Research (CeHRes) roadmap, a five-phase holistic model for the development and implementation of eHealth services [8]. Stakeholder engagement and participation is considered vital throughout the whole process to ensure that an eHealth service fits with stakeholder needs, values and the goals they hope to realize with this service. While service modelling is not an alternative to the CeHRes roadmap, we believe it can be a good addition. Within the CeHRes roadmap, the involvement of stakeholders is heavily based on collecting requirements for the system [36] and not so much on the service process. And while (functional) requirements are considered key output in the CeHReS roadmap, in service modelling they are not.

### Lessons learned

An iterative service model development process, in which conceptual models are presented via scenarios and/or low-fidelity prototypes of the eHealth application allows for thorough integration of the service model with the technical development. In this, one does not need to wait for a fully functioning system. The use of storyboards and low-fidelity prototypes are very sufficient for gathering valuable insights and feedback [52]. Our study illustrates how service model design and system development constantly learn from and influence each other. This lesson most clearly manifests itself in our decision to develop two service models. While our starting point was to develop one service model for one eHealth application, we ended up with two variations of the SALSA service, each with its own service model. This differentiation allows for better alignment with the needs, goals and workflow of the different stakeholders that we aimed to serve.

Specific attention was paid to the workflow of sports trainers and therapists. This was done to overcome potential barriers of implementation for the SALSA service. Various studies [53–55] highlight that one of the key elements in implementation of eHealth is to make sure that the system is adapted to, or creates minimal disruption to the local context and workflow of health professionals. By basing the service model process on the workflows of health professionals and trainers, one can lower this potential implementation barrier.

### Limitations

A common issue with this type of study is to bring all stakeholders, especially clinical stakeholders (like GPs and nurses), together for an in-depth qualitative session, such as an interview or focus group. This study is no exception. While GP’s and medical specialists were in the short list of salient stakeholders, they were not able to participate in the stakeholder focus group. This means that the involvement of GP’s must become clear during the implementation process, instead of the design process. Second, the development of the SALSA service model hinged heavily on input from Dutch stakeholders. Given the fact that each country is different in terms of culture, health prevention programs and setup of medical care, it is difficult to implement a service model, developed in one country, to another country without adaptations. The main lessons from our article, therefore, are on the methodological front, while the results we reported (the (interim) service models) mainly serve an illustrative purpose.

## Conclusions

In this article, we have attempted to create an empirical overview of the service development process for an eHealth service. Service modelling is becoming common practice in commercial eHealth development, but scientific documentation in order to enable further learning for the community is scarce. We hope that our practical discussion of the development of the SALSA service models will inspire other eHealth developers to take both end-user and stakeholder input into account. To elicit their values and needs, and to document how an eHealth application can be used in practice, by whom, where and with what goal. We are sure that including these activities in common eHealth development approaches will increase their quality and chances of success in the real world.

## Supplementary Information

**Additional file 1.** Appendix A: Personas. Appendix B: Illustrations from service model story board.

## Data Availability

Not available.

## Abbreviations

AAL: Active Assisted Living
CBS: Central Bureau for Statistics
CeHRes: Centre for eHealth Research
CVD: Cardiovascular diseases
GP: General practitioner
PT: Physiotherapist
SALSA: Supporting an Active Lifestyle for Seniors through an innovative App-based system for Fitness and Physiotherapy
SES: Socio-Economic Status
UCD: User-centred design

## References

[1] Varsi C, Solberg Nes L, Kristjansdottir OB, Kelders SM, Stenberg U, Zangi HA, Børøsund E, Weiss KE, Stubhaug A, Asbjørnsen RA, Westeng M, Ødegaard M, Eide H (2019). Implementation strategies to enhance the implementation of eHealth programs for patients with chronic illnesses: realist systematic review. J Med Internet Res.

[2] Ahmed B, Dannhauser T, Philip N (2019). A systematic review of reviews to identify key research opportunities within the field of eHealth implementation. J Telemed Telecare.

[3] Geissbuhler A (2013). Lessons learned implementing a regional health information exchange in Geneva as a pilot for the Swiss national eHealth strategy. Int J Med Inform.

[4] May C, Finch T (2009). Implementing, embedding, and integrating practices: an outline of normalization process theory. Sociology..

[5] Mitchell RK, Agle BR, Wood DJ (1997). Toward a theory of stakeholder identification and salience. Acad Manag Rev.

[6] Mantzana V, Themistocleous M, Irani Z, Morabito V (2007). Identifying healthcare actors involved in the adoption of information systems. European Journal of Information Systems.

[7] Hyder A, Puvanachandra P, Bloom G, Sundaram S, Mahmood S, Iqbal M (2010). Stakeholder analysis for health research: case studies from low- and middle-income countries. Public Health.

[8] van Gemert-Pijnen JEWC, Nijland N, van Limburg M, Ossebaard HC, Kelders SM, Eysenbach G (2011). A holistic framework to improve the uptake and impact of eHealth technologies. J Med Internet Res.

[9] Kidholm K, Ekeland AG, Jensen LK, Rasmussen J, Pedersen CD, Bowes A, Flottorp SA, Bech M (2012). A model for assessment of telemedicine applications: mast. Int J Technol Assess Health Care.

[10] Huisin’t Veld RM, Widya IA, Bults RG, Sandsjö L, Hermens HJ, Vollenbroek-Hutten MM (2010). A scenario guideline for designing new teletreatments. J Telemed Telecare.

[11] LeRouge C, Ma J, Sneha S, Tolle K (2013). User profiles and personas in the design and development of consumer health technologies. Int J Med Inform.

[12] Friedman B, Kahn PH, Borning A. Value sensitive design and information systems. In: The Handbook of Information and Computer Ethics. 2009.

[13] Breeman LD, Keesman M, Atsma DE, Chavannes NH, Janssen V, van Gemert-Pijnen L (2021). A multi-stakeholder approach to eHealth development: Promoting sustained healthy living among cardiovascular patients. Int J Med Inform.

[14] Radnor Z, Osborne SP, Kinder T, Mutton J (2014). Operationalizing co-production in public services delivery: the contribution of service blueprinting. Public Manag Rev.

[15] Bitner MJ, Ostrom AL, Morgan FN, Bitner MJ, Ostrom AL, Morgan FN (2008). Service blueprinting: a practical technique for service innovation. Calif Manag Rev.

[16] Shostack GL. Designing services that deliver. Harv Bus Rev. (84115).

[17] Zeithaml V, Bitner M (2000). Services marketing.

[18] Shostack GL (1987). Service Positioning through Structural Change. J Mark.

[19] van Meeuwen DP, van Walt Meijer QJ, Simonse LW (2015). Care models of eHealth services: a case study on the Design of a Business Model for an online Precare service. JMIR Res Protoc.

[20] Kijl B, Nieuwenhuis LJM, Huisin’t Veld RMHA, Hermens HJ, MMR V-H. Deployment of e-health services - A business model engineering strategy. J Telemed Telecare. 2010;16, 344. 10.1258/jtt.2010.006009 20798429

[21] Nam S, Ha C, Lee H (2018). Redesigning in-flight service with service blueprint based on text analysis. Sustainability..

[22] Lee E (2017). A service design thinking approach for stakeholder-centred ehealth. Stud Health Technol Inform.

[23] Van Velsen L, Illario M, Jansen-Kosterink S, Crola C, Di Somma C, Colao A (2015). A community-based, technology-supported health service for detecting and preventing frailty among older adults: a participatory design development process. J Aging Res.

[24] Van Velsen L, Van Weering MD, Luub F, Kemperman A, Ruis M, Urlings J (2019). Travelling with my soulmate: Participatory design of an mHealth travel companion for older adults. ICT4AWE 2019 - Proc 5th Int Conf Inf Commun Technol Ageing Well e-Health.

[25] Ronzi S, Orton L, Pope D, Valtorta NK, Bruce NG (2018). What is the impact on health and wellbeing of interventions that foster respect and social inclusion in community-residing older adults? A systematic review of quantitative and qualitative studies. Syst Rev.

[26] van Velsen L, van Gemert-pijnen L, Nijland N, Beaujean D, van Steenbergen J (2012). Personas: The Linking Pin in Holistic Design for eHealth. Proc 4th Int Conf eHealth, Telemedicine, Soc Med.

[27] Etiken I, Musa SA, Alkassim RS (2016). Comparison of convenience sampling and purposive sampling. Am J Theor Appl Stat.

[28] Nakkeeran N, Zodpey SP (2012). Qualitative research in applied situations: strategies to ensure rigor and validity. Indian J Public Health.

[29] Ritchie J, Spencer J, Bryman A, Burgess R (1993). Qualitative Data Analysis for Applied Policy Research.

[30] Gale NK, Health G, Cameron E, Rashid S, Redwood S (2013). Using the framework method for the analysis of qualitative data in multi-disciplinary health research. BMC Med Res Methodol.

[31] Srivastava A, Thomson SB (2009). Framework analysis: a qualitative methodology for applied policy research. JOAAG..

[32] Tong A, Sainsbury P, Craig J (2007). Consolidated criteria for reporting qualitative research ( COREQ ): a 32-item checklist for interviews and focus groups. Int J Qual Health Care.

[33] Central Bureau for Statistics (2020). Persoonskenmerken, Gezondheid en zorggebruik.

[34] Central Bureau for Statistics (2020). Bevolking; geslacht, leeftijd en burgerlijke staat, 1 januari.

[35] van Woezik AFG, Braakman-Jansen LMA, Kulyk O, Siemons L, van Gemert-Pijnen JEWC (2016). Tackling wicked problems in infection prevention and control: a guideline for co-creation with stakeholders. Antimicrob Resist Infect Control.

[36] Van Velsen L, Wentzel J, Van Gemert-Pijnen JEWC (2013). Designing ehealth that matters via a multidisciplinary requirements development approach. J Med Internet Res.

[37] Van Velthoven MH, Cordon C (2019). Sustainable adoption of digital health innovations: perspectives from a stakeholder workshop. J Med Internet Res.

[38] Lee ML, Dey AK (2012). Embedded assessment of aging adults: a concept validation with stakeholders. 2010 4th Int Conf Pervasive Comput Technol Healthc.

[39] Yu E, Sangiorgi D (2018). Service design as an approach to implement the value Cocreation perspective in new service development. J Serv Res.

[40] Trischler J, Pervan SJ, Kelly SJ, Scott DR (2018). The value of Codesign: the effect of customer involvement in service design teams. J Serv Res.

[41] Fan C, Forlizzi J, Dey A (2012). Considerations for technology that support physical activity by older adults.

[42] van Velsen L, Broekhuis M, Jansen-Kosterink S, Op den Akker H (2019). Tailoring persuasive electronic health strategies for older adults on the basis of personal motivation: web-based survey study. J Med Internet Res.

[43] Hossain LN, Tudball J, Franco-Trigo L, Durks D, Benrimoj SI, Sabater-Hernández D (2018). A multilevel stakeholder approach for identifying the determinants of implementation of government-funded community pharmacy services at the primary care level. Res Soc Adm Pharm.

[44] Yoo D. Stakeholder tokens: A constructive method for value sensitive design stakeholder analysis. In: DIS 2017 Companion - Proceedings of the 2017 ACM Conference on Designing Interactive Systems. p. 2017.

[45] Burnay C, Horkoff J, Maiden N (2016). Stimulating Stakeholders’ Imagination: New Creativity Triggers for Eliciting Novel Requirements. Proceedings - 2016 IEEE 24th international requirements engineering conference, RE 2016.

[46] Lusch RF, Nambisan S (2015). Service innovation: a service-dominant logic perspective. MIS Q Manag Inf Syst.

[47] Grenha Teixeira J, Patrício L, Huang KH, Fisk RP, Nóbrega L, Constantine L (2017). The MINDS Method: Integrating Management and Interaction Design Perspectives for Service Design. J Serv Res.

[48] Bitner MJ, Booms BH, Tetreault MS (1990). The Service Encounter: Diagnosing Favorable and Unfavorable Incidents. J Mark.

[49] Patrício L, Fisk RP, Falcão E, Cunha J (2008). Designing multi-interface service experiences: the service experience blueprint. J Serv Res.

[50] Norman DA (1986). User Centered System Design User Centered System Design.

[51] Ferris TLJ (2004). User-centered design: an integrated approach. IEEE Trans Prof Commun.

[52] Van Velsen L, Evers M, Bara CD, Op Den Akker H, Boerema S, Hermens H (2018). Understanding the acceptance of an ehealth technology in the early stages of development: An end-user walkthrough approach and two case studies. J Med Internet Res.

[53] Ross J, Stevenson F, Lau R, Murray E (2016). Factors that influence the implementation of e-health: a systematic review of systematic reviews (an update). Implement Sci.

[54] Granja C, Janssen W, Johansen MA (2018). Factors determining the success and failure of ehealth interventions: systematic review of the literature. J Med Internet Res.

[55] Abbott PA, Foster J, Marin Hde F, Dykes PC (2014). Complexity and the science of implementation in health IT-knowledge gaps and future visions. Int J Med Inform.

