# Supplementary material for: Designing a stakeholder-inclusive service model for an eHealth service to support older adults in an active and social life
Source: BMC Health Serv Res. 2021 Jul 5;21:654. doi: 10.1186/s12913-021-06597-9 (PMC8256482; doi:10.1186/s12913-021-06597-9)
Supplement: Supplementary file 1 — Additional file 1. Appendix A: Personas. Appendix B: Illustrations from service model story board. [file 12913_2021_6597_MOESM1_ESM.docx]

Supplementary file: Appendix

**Designing a stakeholder-inclusive service model for an eHealth service to support older adults in an active and social life**

Marijke Broekhuis* ^a,b^ ([m.broekhuis@rrd.nl](mailto:m.broekhuis@rrd.nl)), Marit Dekker-van Weering^c^, Cheyenne Schuit^d^ ([schuit@innoboost.nl](mailto:schuit@innoboost.nl)), Stefan Schürz ^e^ ([stefan.schuerz@lifetool.at](mailto:stefan.schuerz@lifetool.at)) and Lex van Velsen ^a,b^ ([l.vanvelsen@rrd.nl](mailto:l.vanvelsen@rrd.nl))

^a^ Roessingh Research and Development, eHealth group, Roessinghsbleekweg 33b, 7522AH Enschede, the Netherlands

^b^ Biomedical Signals and Systems, Faculty of Electrical Engineering, Mathematics and Computer Science (EEMCS), University of Twente, Enschede, the Netherlands

^c^ TSN Thuiszorg Groningen

^d^ National Foundation for the Elderly, Smallepad 30e, 3811 MG Amersfoort, the Netherlands

*^e^ LIFEtool gemeinnützige GmbH, Linz, Austria*

* Corresponding author

# Appendix A: Personas

| **Persona** | **Demographics** | **Behaviours** | **Needs and goals** |
| --- | --- | --- | --- |
| 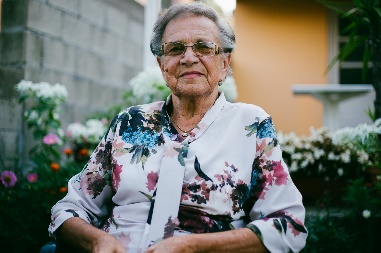  Jenny  *(Photo by Damir Bosnjak on Unsplash)* | - 72 years old - Married, 2 kids - Lives in small village - Low digital literacy | Jenny is strongly focused on her family and calls her kids regularly. She likes gardening and baking. | She has problems with her hip and back and needs physiotherapy. She wants to become more active and adopt a healthier lifestyle |
| 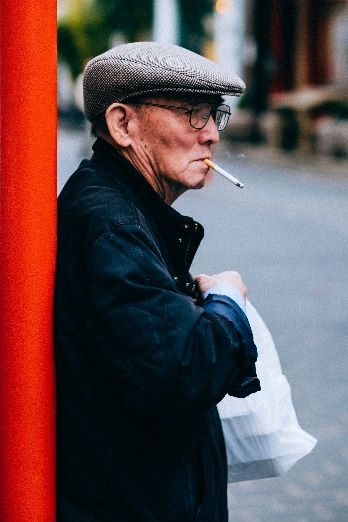  Marcus  *(Photo by Oliver Cole on Unsplash)* | - 75 years old - Divorced, 1 son - Lives in small village - Low digital literacy | Marcus does not like to exercise. He is a big soccer fan and spends large parts of the day watching TV. He is a heavy smoker and is suspicious of doctors and therapists. | Marcus has COPD for some time now. He is physically limited but wants to remain independent. He also wants to have more social contacts. |
| 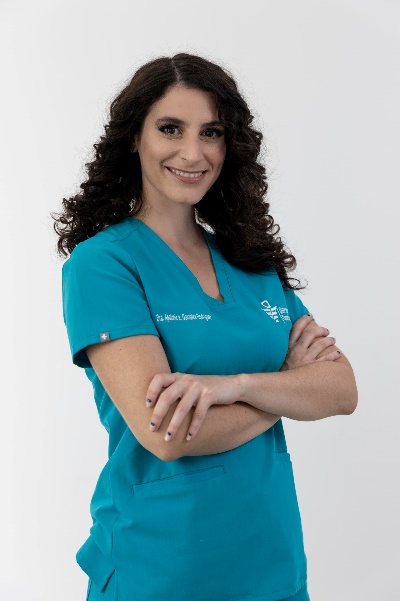  Wendy  *(Photo by Brian Mercado on Unsplash)* | - 28 years old - Physiotherapist - Single - Lives and works in the city (own practice) - High digital literacy | Wendy loves her job and is always open to new ideas. She is a sporty person and likes travelling and dancing. | She is always looking for ways to improve therapy and believes ICT solutions could help to improve the workflow or to improve motivation and compliance of her clients. |

# Appendix B: Illustrations from service model story board
